# Resveratrol interferes with AKT activity and triggers apoptosis in human uterine cancer cells

**DOI:** 10.1186/1476-4598-5-45

**Published:** 2006-10-17

**Authors:** Émilie Sexton, Céline Van Themsche, Kim Leblanc, Sophie Parent, Pascal Lemoine, Eric Asselin

**Affiliations:** 1 Département de Chimie-Biologie, Groupe de Recherche en Biopathologies Cellulaires et Moléculaires, Université du Québec à Trois-Rivières, C.P. 500, Trois-Rivières, Québec, G9A 5H7, Canada

## Abstract

**Background:**

Endometrial cancer is the fourth most prominent cancer among all feminine cancers in the Western world. Resveratrol, a natural anti-oxidant found in red wine emerging as a novel anticancer agent, exerts antiproliferative and pro-apoptotic activity in various cancer cell types, but its effect on uterine cancer cells is poorly understood. At the molecular level, resveratrol has been reported to inhibit cyclooxygenase (COX) expression and/or activity; in endometrial cancer cells, COX-2 is overexpressed and confers cellular resistance to apoptosis. The aim of the present study was to determine if resveratrol could exert anti-proliferative and pro-apoptotic activity over uterine cancer cells upon inhibition of COX-2 expression and/or activity. Six different human uterine cancer cell lines were used as a model (HeLa, Hec-1A, KLE, RL95-2, Ishikawa and EN-1078D).

**Results and discussion:**

High-dose of resveratrol triggered apoptosis in five out of six uterine cancer cell lines, as judged from Hoechst nuclear staining and effector caspase cleavage. In accordance, uterine cancer cell proliferation was decreased. Resveratrol also reduced cellular levels of the phosphorylated/active form of anti-apoptotic kinase AKT. Endogenous COX-2 protein levels were decreased, concomitant with a decrease in production of COX metabolites PGE2 and PGF2α, in each uterine cancer cell line expressing detectable levels of COX-1 and/or COX-2 in presence of resveratrol. Although COX expression was identified as a target of resveratrol in uterine cancer cells, inhibition of COX activity or exogenously added PGE2 did not modulate the effect of resveratrol on cellular proliferation.

**Conclusion:**

High-dose of resveratrol exerts tumoricidal activity over uterine cancer cells and regulates COX expression. In these cells, resveratrol would not directly target COX activity, but possibly other enzymes involved in prostaglandin synthesis that act downstream of the COXs.

## Background

Resveratrol (3,4',5-trihydroxy-trans-stilbène) is a natural phytoalexin present in grape skins and consequently, in red wines and grape juices [1,2]. When used at low concentrations resveratrol has cytoprotective activity, which is mostly attributed to its antioxidant properties [3]. However, when administered at higher doses, resveratrol possesses anti-cancer activity by interfering with different cellular events associated with initiation, promotion and progression of multi-stage carcinogenesis [4]. The chemopreventive activity of resveratrol has notably been demonstrated in breast [5] and prostate [6] cancers. The anticancer potential of resveratrol in endometrial carcinoma, however, remains unknown.

Endometrial carcinoma is the fourth type of cancer with respect to incidence rates and is the leading type of gynecological cancer in the Western world. In mutated-PTEN endometrial tumors, which constitute around 50% of all tumors [7], AKT is constitutively phosphorylated [8] and its activity notably leads to up-regulation of COX-2 [8,9]. COX-2 up-regulation has also been found in other types of cancers such as cervix [10], prostate [11] and breast [12], where it has been shown to play a role in tumorigenesis [13-15]. In endometrial carcinoma cells, expression of COX-2 notably confers resistance to apoptosis [8]. Similar to COX-2, COX-1 is up-regulated in different carcinomas [16,17] and plays a role in tumorigenesis [18,19]; COX-1 is also present in endometrial tumors [20]. COX-1 and COX-2 are the rate-limiting enzymes involved in the biosynthesis of prostaglandins (PGs). PGE_2_, their major metabolite, mediates its biological function through interaction with the G protein-coupled receptors [21], namely EP1, EP2, EP3 and EP4. The EP1 receptor activates phospholipase C and the mobilization of the inositol triphosphate pathway; the EP2 and EP4 receptors are coupled to the adenylate cyclase and activate the cAMP/protein kinase A pathway, and the EP3 receptor inhibits adenylate cyclase and activates the phospholipase C [22]. The synthesis of PGE_2 _would be key elements in the pathophysiology of different cancers [23,24]; notably, PGE2 exerts immunosuppressive [25,26] as well as pro-angiogenic [27,28] activities.

The malignant uterus thus constitutes a potential target of resveratrol, given that resveratrol has been shown to inhibit COX-2 transcription and activity [29,30], and that COX-2 is overexpressed in a large proportion of endometrial tumor cells lines [8] and a majority of human endometrial carcinoma specimens [20,31]. In light of our previous findings that the presence of COX-2 promotes the survival of endometrial tumor cells [8], we hypothesized that resveratrol could exert tumoricidal activity over uterine cancer cells upon inhibition of COX-2 expression and/or activity. In addition, recent reports by Szewczuk and colleagues suggest that resveratrol can also inhibit the enzymatic activity of COX-1 [32-34], which is present in many endometrial tumor tissues [20]; it is therefore possible that resveratrol would also modulate COX-1 expression and/or activity in uterine cancer cells. We have undertaken the present study to determine how resveratrol influences survival/apoptosis, and COX expression and activity in uterine cancer cells. Our results showed that resveratrol can induce apoptosis in five of the six uterine cancer cell lines studied, triggering effector caspase cleavage and decreasing phospho-Akt levels. Concomitant reduction in COX-2 levels and prostaglandins production was observed.

## Results

### Resveratrol triggers apoptosis in uterine cancer cells

**Figure 1:**
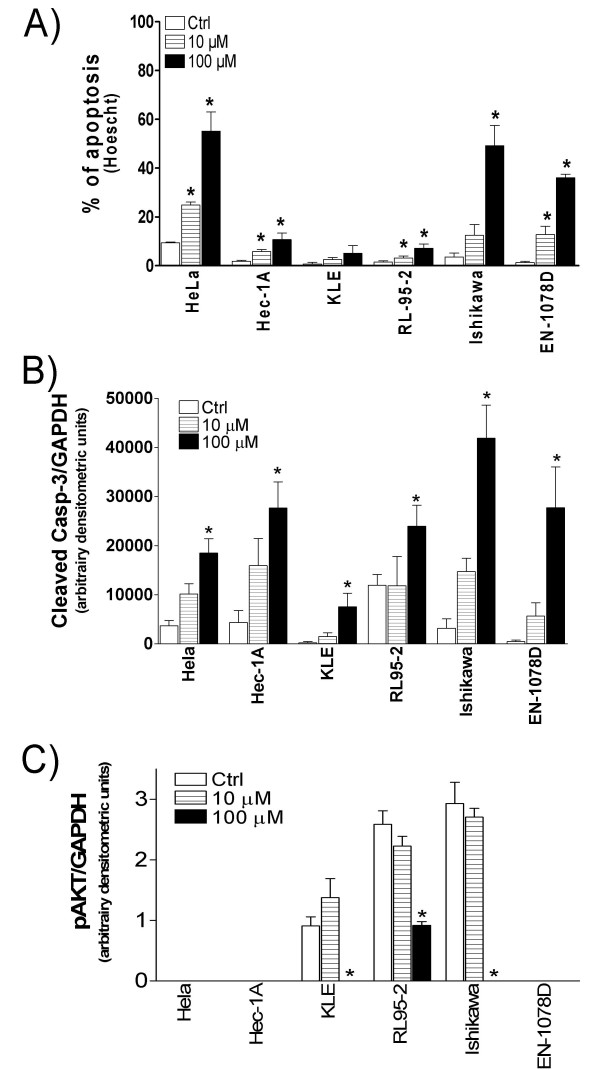
Effect of resveratrol (0 (control or Ctrl), 10 and 100 μM) on apoptosis induction in HeLa, HEC-1A, KLE, RL95-2, Ishikawa and EN-1078D cell lines, after a period of 48 hours. **A) **Hoescht nuclear staining was performed, and apoptotic cells were counted under a microscope. Results, presented as a percentage of apoptotic cells to the total cell count, are mean +/- SEM of three independent experiments. **B) **Cleavage/activation of caspases-3 was monitored by Western blot. GAPDH was used as a loading control; results are mean +/- SEM of three independent experiments. **C) **The effect of resveratrol of the levels of pAKT was determined by western blot analysis. GAPDH was used as a loading control. Results are mean +/- SEM of at least two independent experiments. *p < 0.05 compared to control cells (Ctrl).

Resveratrol has been shown to trigger apoptosis in a few tumor cell types [35,36], and we examined whether resveratrol could also exert pro-apoptotic activity in uterine cancer cells. Hoescht staining revealed that a low dose of 10 μM resveratrol was sufficient to increase apoptosis in HeLa, EN-1078D, HEC-1A and RL95-2 (Fig. 1A), whereas a higher dose of 100 μM resveratrol was required to increase significantly the apoptotic index of Ishikawa cells. In accordance, Western blot analysis of caspase-3 cleavage showed that resveratrol increased the levels of the active form of caspase-3 in the six cancer cell lines tested (Fig. 1B). These results indicate that resveratrol can activate apoptotic pathways in uterine cancer cells.

### Resveratrol interferes with AKT survival pathway in uterine cancer cells

Resveratrol has been reported to inhibit AKT activation [37,38], and we have previously demonstrated that the activity of AKT directly modulated survival/apoptosis in uterine cancer cells [8]. We have thus determined whether resveratrol interfered with AKT phosphorylation/activation in uterine cancer cell lines. Intracellular levels of phosphorylated (active) AKT were not modified by resveratrol at 10 μM, but we found that a dose of 100 μM of resveratrol decreased the levels of phosphorylated AKT in the three cell lines expressing constitutive levels of the kinase (KLE, RL95-2 and Ishikawa), while it had no observable effect on the other cell lines (Fig. 1C), indicating that resveratrol interferes with AKT activity in uterine cancer cells.

### Resveratrol modifies the proliferating activity of uterine cancer cells

**Figure 2:**
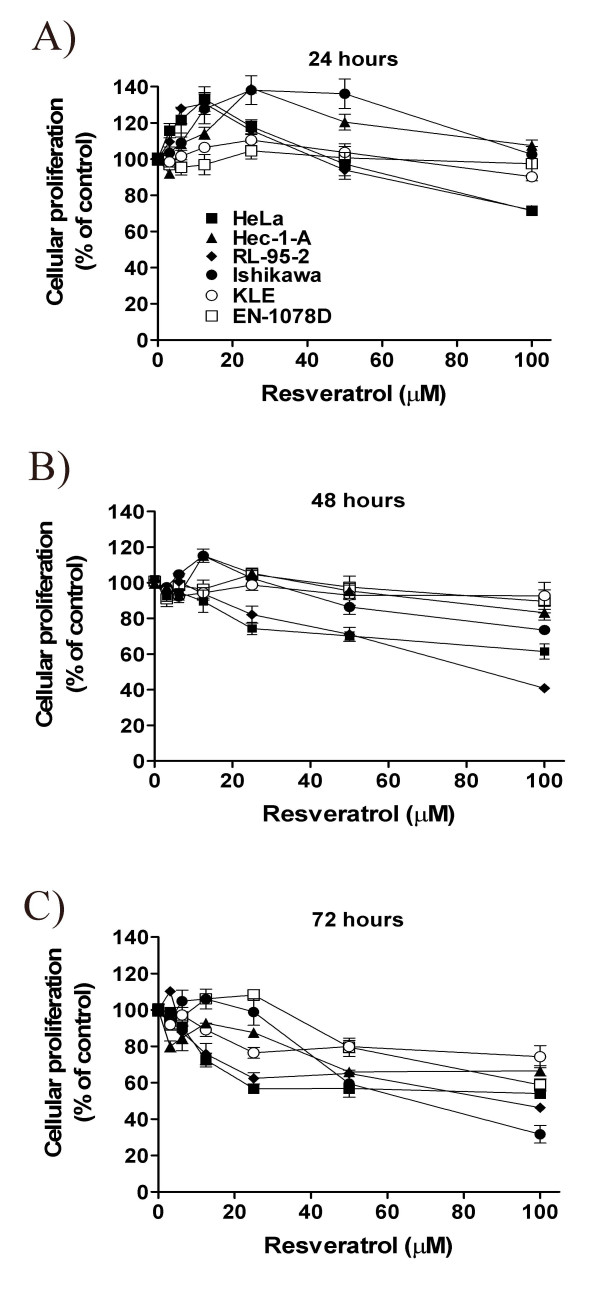
The effect of resveratrol (0 (control or Ctrl), 3.125, 6.25, 12, 25, 50 and 100 μM) on cellular proliferation in HeLa (■), HEC-1A (▲), RL95-2 (◆), Ishikawa (●), KLE (○) and EN-1078D (□) cells for a period of **A) **24 hours, **B) **48 hours and **C) **72 hours was determined by MTT proliferation assay. Results, which are presented as a percentage of proliferation of treated cells compared to control (untreated) cells, are mean +/- SEM of three independent experiments, each performed in duplicates.

Resveratrol has been shown to exert antiproliferative activity on particular tumor cell types [5,6,39], and we have determined whether it could also modulate the growth properties of uterine cancer cells. To this aim, MTT assay was performed on uterine cancer cells that had been treated with increasing concentrations of this natural compound. We found that resveratrol indeed modified the proliferating activity of uterine cancer cells, and its effect was biphasic: cellular proliferation was transiently increased at low doses (3.125–12 μM), whereas higher concentrations (50–100 μM) of resveratrol rather caused time-dependent decrease of proliferating activity (Fig. 2). These results indicate that resveratrol modifies the proliferating activity of uterine cancer cells.

### Resveratrol regulates COX expression in uterine cancer cells

**Figure 3:**
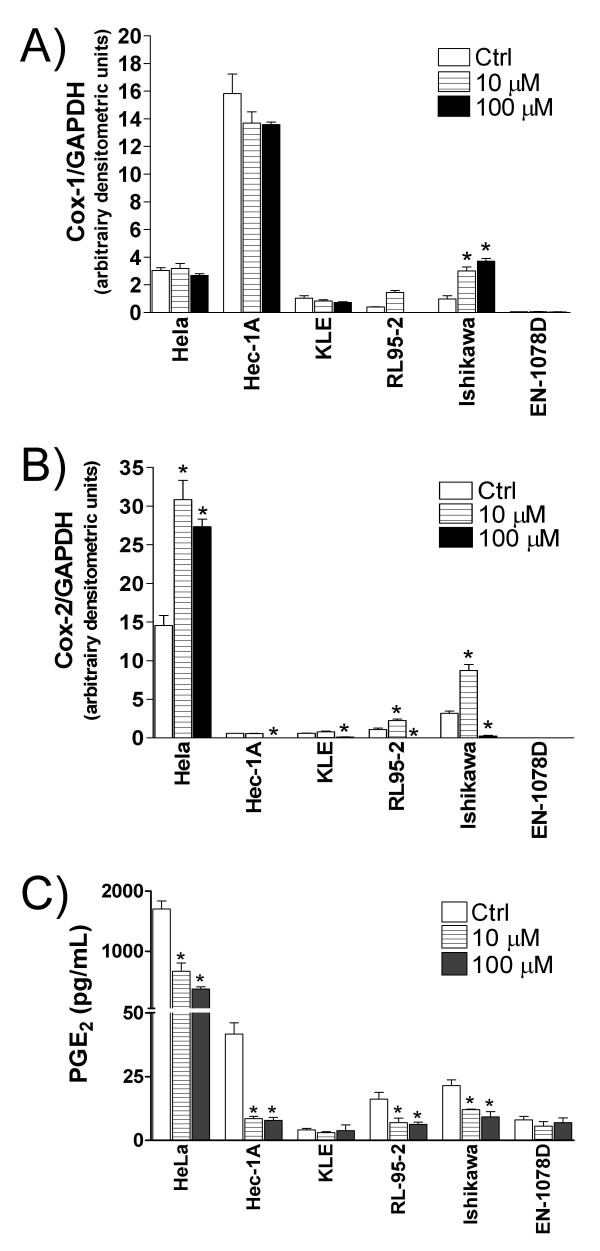
The effect of resveratrol of the levels of **A) **COX-1 and **B) **COX-2 was determined by western blot analysis. GAPDH was used as a loading control. Results are mean +/- SEM of at least two independent experiments. *p < 0.05 compared to control. **C) **The effect of a 48h-treatment with resveratrol (0, 10 and 100 μM) on PGE_2 _production by HeLa, HEC-1A, KLE, RL95-2, Ishikawa and EN-1078D cell lines was determined using EIA assay. Data represent the mean ± SEM of 4 independent experiments. *p < 0.05 compared to control (Ctrl).

It has been reported that resveratrol modifies COX levels in varying cell types [30,40]. In order to determine the effect of resveratrol on the expression of COX enzymes in uterine cancer cells, we have treated these cells with a low (10 μM) and a high (100 μM) dose of resveratrol, and subsequently measured COX levels using western blot. We found that COX-1 was regulated by resveratrol in only one of the six cell lines tested: in Ishikawa cells, resveratrol caused a dose-dependent increase in COX-1 expression (Fig. 3A). In contrast, resveratrol had various effects on COX-2 levels, depending on the dose and the cell line that were used. In cells expressing high constitutive COX-2 levels (Hela, RL95-2 and Ishikawa), 10 μM resveratrol further increased these levels; however, a dose of 100 μM resveratrol rather decreased COX-2 levels in all cell lines tested except for Hela cells (Fig. 3B). We also evaluated whether resveratrol had an effect on the production of prostaglandins by uterine cancer cells. First, our results showed that these cells constitutively produce varying levels of PGE2, a major metabolite of COX enzymes, which correlate well with endogenous COX levels (Fig. 3A–C). Then, resveratrol had no effect on the production of PGE2 by KLE and EN-1078D cell lines, which do not express detectable levels of any of the COX enzymes (Fig 3C); noteworthy, in the four cells lines containing endogenous levels of COX-1 and/or COX-2 enzymes (Hela, Hec-1A, RL95-2 and Ishikawa), resveratrol decreased the production of PGE2 (Fig. 3C), irrespective of its effect on COX levels which varied depending on the dose used (Fig. 3A–B). Moreover, the levels of PGF2α, another metabolite of COX activity, were also decreased by resveratrol (data not shown). Collectively these results indicate that in addition to interfering with COX expression, resveratrol inhibits the production of prostaglandins by uterine cancer cells.

### COX activity might not be involved in resveratrol-induced cell proliferation

**Figure 4:**
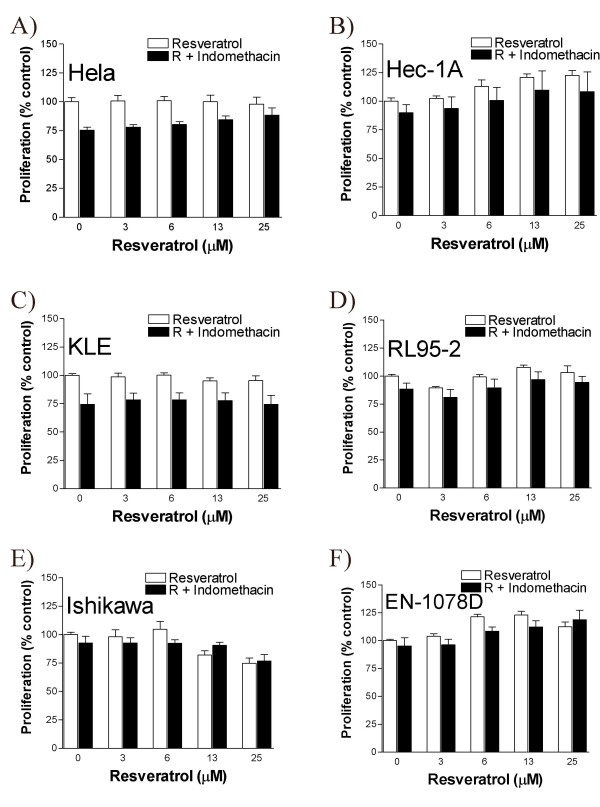
Effect of resveratrol (0, 3.125, 6.25, 12, 25, 50 and 100 μM), in the presence or absence of 5 μM indomethacin for a period of 48 hours, on cellular proliferation in A) HeLa, B) HEC-1A, C) KLE, D) RL95-2, E) Ishikawa and F) EN-1078D cells as determined by the MTT proliferation assay. Data represent the mean ± SEM of 6 independent experiments.

At low dose resveratrol increases the proliferation (Fig. 2), and upregulates COX levels (Fig. 3B) in multiple uterine cancer cell lines. Since increased COX-2 levels has been shown to promote survival of uterine cancer cells [8], we hypothesized that elevated COX enzymes levels could promote the proliferating activity of these cells and could be involved in the positive effect of low-dose resveratrol on cellular proliferation. We found that indomethacin, which inhibits the activity of both COX-1 and COX-2 enzymes, decreased basal proliferation of Hela and KLE cell lines (Fig. 4); we also observed that NS-398, a selective in inhibitor of COX-2 activity, decreased cellular proliferation of RL95-2, Ishikawa and EN-1078D (data not shown), suggesting that COX enzymes are involved in basal proliferating activity of uterine cancer cells. In a combined treatment however, indomethacin did not block the positive action of low-dose of resveratrol on cellular proliferation (Fig. 4). Similarly, NS-398 could not interfere with the effect of low-dose of resveratrol on cellular proliferation (data not shown), indicating that the increase of cellular proliferation following exposure to low-dose resveratrol does not result from increased COX activity. Noteworthy, indomethacin and NS-398 did not mediate the anti-proliferative effect of high-dose resveratrol on uterine cancer cell proliferation (data not shown), suggesting that resveratrol does not decrease uterine cancer cell proliferation by inhibiting COX activity.

### PGE_2 _does not interfere with resveratrol antiproliferative activity

**Figure 5:**
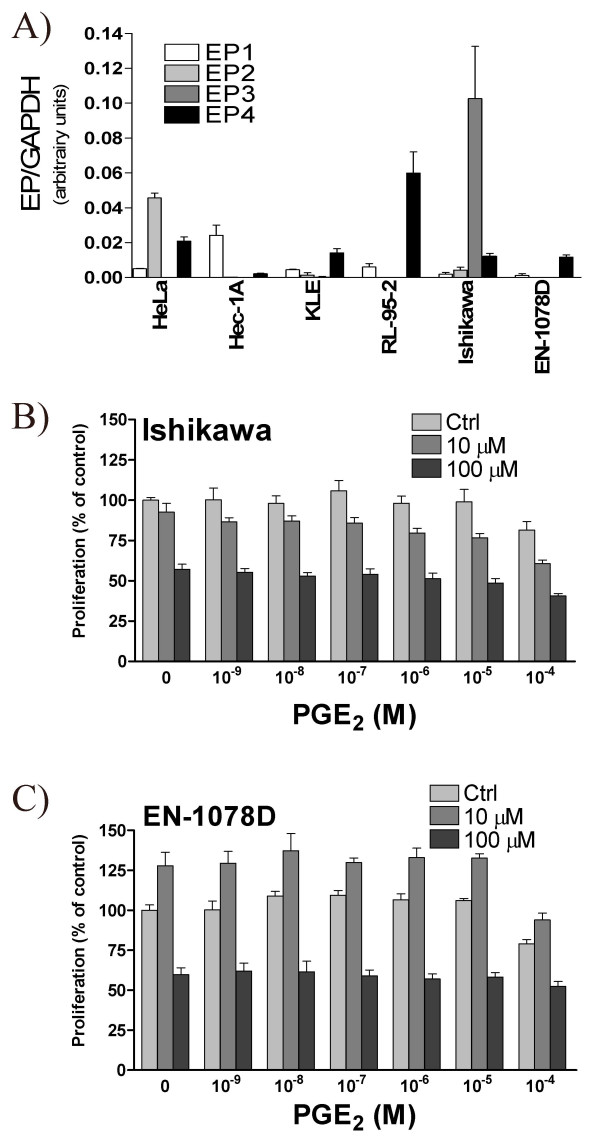
**A) **The levels of EP1 to EP4 receptors mRNA were determined in HeLa, HEC-1A, KLE, RL95-2, Ishikawa and EN-1078D cell lines by quantitative real-time PCR. GAPDH was used as control to correct for loading. Data represent mean ± SEM of 3 independent experiments. **B) **The effect of PGE_2 _(0, 10^-9^, 10^-8^, 10^-7^, 10^-6^, 10^-5 ^and 10^-4 ^M), in the presence or absence of 10 or 100 μM resveratrol, was evaluated on cellular proliferation in B) Ishikawa and C) EN-1078D cells, for a period of 48 hours, using the MTT proliferation assay. Data represent the mean ± SEM of 6 independent experiments. Ctrl: untreated control.

Since high-dose of resveratrol decreases PGE2 production in uterine cancer cells (Fig. 3C), concomitant with a decrease in cellular proliferation (Fig. 2), we hypothesized that PGE2 could promote cellular proliferation and counteract the anti-proliferative effect of high resveratrol dose. First we assessed the presence of PGE2 receptors on the six uterine cancer cells lines, and observed that more than one EP receptor were expressed by each cell line (Fig. 5A). EP1 was more abundant in HEC-1A cells, while EP2 was expressed at higher levels in HeLa cells; EP3 mRNA was found to be elevated in Ishikawa cell line compared with the five other cancer cell lines tested and RL95-2 expressed the highest levels of EP4 (Fig. 5A). We next chose as a model Ishikawa and EN-1078D; Ishikawa cells constitutively express COX enzymes, the levels of which are modulated by resveratrol, and conversely EN-1078D cells express no detectable levels of COX, even in the presence of resveratrol (Fig. 3A–B). The two cell lines were incubated with increasing concentrations of PGE2, alone or in combination with resveratrol (10 and 100 μM). We found that PGE_2 _alone did not promote cellular proliferation in these two cell lines (Fig. 5B–C); in addition, exogenous PGE2 was not able to counteract the effect of resveratrol on the proliferation of Ishikawa and EN-1078D cell lines (Fig. 5B–C).

## Discussion

Resveratrol, a phytoestrogen found in food products such as grape and wine, produces various physiological effects. At low concentrations normally occurring in food, resveratrol has been shown to exert neuroprotective effects [41], as well as beneficial effects on the cardiovascular system [42,43]. These effects are mostly attributed to its anti-oxidant properties. More recently, resveratrol has been evaluated for its health benefits in other medical areas, such as oncology; resveratrol indeed possesses anticancer activity when administered at higher, non-physiological doses. In these conditions, resveratrol inhibits the proliferation and induces apoptotic cell death in multiple cancers cell types *in vitro *[6,44,45]; moreover, in animal models of cancer, resveratrol has been shown to inhibit angiogenesis and delay tumor growth [46], to impede carcinogenesis [4,47], and to reduce experimental metastasis [48].

The malignant uterus constitutes a potential target for resveratrol. First, COX-2 is overexpressed in a large proportion of endometrial tumors [8], and resveratrol has been shown to inhibit COX expression and activity in a variety of cell types [32,49]. Second, resveratrol has the capacity to bind to the estrogen receptors α and β [50], and ER-α is notably present and activated in the normal and neoplastic endometrium [51]. Nonetheless, the effects of resveratrol on uterine cancer cells remain largely unknown. We have examined the effect of resveratrol on survival/apoptosis of uterine cancer cells, in relation to its effects on COX expression and activity. To this aim, we have used five cancer lines of the human endometrium (Hec-1A, KLE, RL95-2, Ishikawa and EN-1078D) and one cancer line of the human uterine cervix (HeLa).

In the course of these experiments we have observed that even though high doses (50–100 μM) of resveratrol induce apoptosis in uterine cancer cells, at low doses (up to 25 μM) resveratrol rather transiently increases proliferation in several uterine cancer cell lines. This indicates that resveratrol activates, in a dose-dependent manner, distinct cellular mechanisms in these cells. Our results showed that even though low doses of resveratrol upregulate the levels of COX-1 and/or COX-2 enzymes in uterine cancer cells, COX activity is not involved in the increase of cellular proliferation caused by resveratrol. We have previously demonstrated that Akt kinase was a central element in the survival of uterine cancer cells when exposed to pro-apoptotic agents [52], and it has been reported that resveratrol at concentrations close to 10 μM could increase PI3-K activity and the levels of phosphorylated/active Akt in MCF-7 breast cancer cells [37]. We have found, however, that the levels of phosphorylated Akt were not increased in uterine cancer cells following exposure to low doses of resveratrol, which suggests that resveratrol does not increase the proliferation of uterine cancer cells through Akt regulation. Resveratrol can act as an estrogen agonist or antagonist, depending on the estrogen response element sequence that is present and whether the phytoestrogen binds ERα or ERβ [50]; cells that only express ERβ or that express higher levels of ERα compared to ERβ would be more sensitive to the estrogen agonist activity of resveratrol [50]. Among the six uterus cancer cell lines that have been tested in the present study, Hela presents the highest ratio ERβ to ERα, while EN-1078D (the only cell line expressing ERα in our experimental conditions) has the lowest expression [53]. We previously showed that proliferation of uterine cancer cell lines was increased in response to estrogen [53]. Thus, if resveratrol exerts estrogen agonist effect on ERβ uterine cancer cell lines, it should stimulate proliferation in Hela but not in EN-1078D. Our results showed the opposite, and this suggests that low-dose induction of proliferation by resveratrol is not mediated through the ER pathway.

It had already been reported that resveratrol could decrease the proliferation of Ishikawa cell line [54]. We have obtained similar results with this cell line; in addition, we demonstrate that high-dose resveratrol induces a diminution of cellular proliferation in five other uterus cancer cell lines. Resveratrol also caused a rise in the apoptotic cell death in all uterine cancer cells studied, except in KLE cell line albeit there was an increase in cleaved caspase-3 levels. In this regard, we have measured high levels of XIAP, an endogenous inhibitor of effector caspases such as caspase-3, in KLE cells (data not shown); this could explain why the increase in cleaved caspase-3 levels is not accompanied by an increase in apoptotic index. Noteworthy, this cell line is also resistant to the cytotoxic effects of various chemotherapeutic agents [52]. We have also shown that in all uterus cancer cell lines containing endogenous levels of the phosphorylated/active form of AKT kinase (pAKT), high-dose resveratrol decreased pAKT levels, concomitant with an increase in cleaved caspase-3 levels. Others had also reported that resveratrol can interfere with PI3-kinase activity and Akt phosphorylation [37,55,56] in cancer cells. These results are in agreement with our previous findings that the activity of the PI3-K/Akt pathway is involved in the survival of uterine cancer cells [8].

We show that resveratrol has a dual effect on COX expression and prostaglandin production in uterine cancer cells. Similar to other cell types, where resveratrol up- or downregulates COX levels [30,40,49,57,58], we demonstrate that in uterine cancer cells resveratrol alters COX-2 expression, in a cell- and dose-specific manner. Szewczuk and coworkers have shown that resveratrol can act on COX-1 enzymatic activity [32]; in agreement, we demonstrate that resveratrol inhibits the production of PGE_2 _and PGF2α in all cell lines expressing COX-1 (HeLa, HEC-1A or Ishikawa). However, resveratrol also induced a diminution in the production of PGE_2 _and PGF2α in RL95-2 cells, which express COX-2 but not COX-1. This leads us to believe that resveratrol can act on a target other than the COX-1 protein; in this regard, another study proposed an effect of resveratrol on both the transcriptional activity of COX-2 gene and COX-2 activity in breast cancer [49]. Accordingly, all uterine cancer cell lines expressing COX-2 (HeLa, RL95-2 and Ishikawa) have seen their PGE2 and PGF2α levels decreased following a treatment with high dose resveratrol, which also decreased COX-2 levels. It should be noted, however, that even if resveratrol increased COX-1 or COX-2 levels, which occurred when the cells were exposed to low doses of resveratrol, PGE2 and PGF2α production were decreased. This suggests that in addition to regulating the expression of COX-2, resveratrol might block the activity of enzymes involved in prostaglandin synthesis, possibly COX enzymes themselves but also downstream prostaglandin synthases. We have used pharmacological inhibitors to examine whether resveratrol directly targets COX activity. Our results have showed that the activity of COX enzymes was involved in basal proliferating activity of multiple uterine cancer cells lines; others had reported similar results with pancreatic cancer cells, which also produce endogenous levels of COXs [59]. It appeared, however, that increase of cellular proliferation following exposure to low-dose resveratrol was not blocked by COX inhibitors indomethacin and NS-398; in addition, indomethacin and NS-398 did not modulate the anti-proliferative effect of high-dose of resveratrol (data not shown), suggesting that resveratrol does not influence cellular proliferation by modulating COX activity in uterine cancer cells. Moreover, the cell line EN-1078D, which does not express detectable levels of COX enzymes, was sensitive to the pro-apoptotic effects of high-dose resveratrol. Taken together, these results suggest that resveratrol does not directly target COX activity but rather, downstream components involved in prostaglandin synthesis such as PGE and PGF synthases.

Bhat and coworkers have reported that resveratrol downregulates ER-α in Ishikawa cell line [60]. In most uterine cancer cell lines ER-α is not likely to be targeted by resveratrol however, since it is rarely constitutively expressed (data not shown). Others have reported that resveratrol antagonizes ER-β at high doses [61]; blockade of ER-β could very well occur in uterine cancer cells which all express endogenous levels of this receptor (data not shown). Noteworthy, in MCF-7 breast and Ishikawa endometrial cancer cell lines, the anti-proliferative effect of resveratrol was shown to be ER-independent [61,62]. It may also be the case for the other uterine cancer cells studied.

It has been reported that resveratrol can interfere with PI3-kinase activity [37]. In uterine cancer cells, the expression of COX-2 is regulated by the activity of the PI3-K/Akt pathway [8,9], and since resveratrol regulates COX-2 expression and the extent of phosphorylation of AKT in cells expressing constitutive phospho-AKT levels, it is possible that PI3-K activity is targeted by resveratrol in these cells. Although specific PI3-K inhibitor LY294002 inhibits the growth of uterine cancer cells [8], preliminary experiments indicate that LY294002 does not block the increase of cellular proliferation triggered by low doses of resveratrol (data not shown), suggesting that PI3-K/AKT pathway is not influenced by resveratrol, at least at low doses. This does not rule out the possibility, however, that high-dose resveratrol inhibits the activity of PI3-K, which could explain the observed decrease in pAKT levels.

## Conclusion

In conclusion, resveratrol can inhibit cell growth and trigger apoptotic cell death in uterine cancer cells expressing COX-1 and/or COX-2, in association with a decrease in COX-2 expression and inhibition of prostaglandin production. Noteworthy, resveratrol can also trigger apoptosis in uterine cancer cells which do not express detectable levels of COX-1 and COX-2, confirming that resveratrol can target other enzymes than COXs. This study on uterine cancer adds to a growing body of evidence identifying resveratrol as a promising anti-cancer agent.

## Methods

### Reagents

Resveratrol, Indomethacin, MTT (3-(4,5-dimethyl-thiazolyl-2)-2,5-diphephenyltetrazolium bromide) and Hoechst 33258 were obtained from Sigma (St. Louis, MO). Prostaglandin E_2 _was obtained from Cayman Chemical (Ann Harbor, MI). DMEM-F12, Mc Coy's PCR primers, FBS and BGS sera were purchased from Invitrogen (Burlington, ON). Cleaved caspase-3 antibodies were obtained from New England Biolabs (Mississauga, ON) and anti-human COX-1 and COX-2 GAPDH were obtained from Cedarlane Laboratories (Hornby, ON). Secondary horse radish peroxidase (HRP)-conjugated anti-rabbit antibody and secondary horse radish peroxidase (HRP)-conjugated anti-mouse antibody were purchased from BioRad (Mississauga, ON).

### MTT proliferation assay

Cells were plated at a density of 2 × 10^4 ^cells/well in 96-well plates 24 hours before the assay. Cells were cultured for 24, 48 and 72 hours in the presence of different concentrations of Resveratrol (0; 3.125; 6.25; 12.5; 25; 50 and 100 μM in 0,1 % DMSO). At the end of the culture period, 10 μl of MTT (5 mg/ml) was added to each well. After 3.5 hours of incubation with MTT, 100 μl of solubilization solution was added (10% SDS in 0.01 M HCl) and the microplate was incubated overnight (37°C, 5% CO_2_). The OD was read with Microplate Manager (ELISA) at 550 nm. MTT was also performed with Resveratrol (0; 3.125; 6.25; 12.5; 25; 50 and 100 μM in 0,1 % DMSO) in the presence or absence of 5 μM (0,05% DMSO) indomethacin (COX inhibitor), or 5 μM PGE_2 _(0, 10^-9^, 10^-8^, 10^-7^, 10^-6^, 10^-5 ^and 10^-4 ^M in 0,1% DMSO).

### Resveratrol treatment

HeLa, HEC-1A, KLE, RL95-2, Ishikawa and EN-1078D cells were plated at a density of 1 × 10^6 ^cells/dish (100 mm × 20 mm) 24 hours before treatment. Cells were treated for 48 hours with resveratrol (0,10 and 100 μM).

### Protein extraction and Western analysis

Cells (both floating and attached) were trypsinized, lysed in lysis buffer (PBS 1X pH 7.4; 1% Nonidet P-40; 0.5% Sodium deoxycholate; 0.1% SDS; Protease Inhibitor Cocktail Tablets (Roche)), frozen at -20°C and thawed three times, and centrifuged (13000 × g, 20 min at 4°C) to remove insoluble material. Supernatant was recovered and stored at -20°C pending analysis. Protein content was determined with the Bio-Rad DC Protein Assay. Protein extracts (50 μg) were heated (95°C, 3 min), resolved by 10% SDS-Polyacrylamide gel electrophoresis (PAGE) and electro-transferred to nitrocellulose membranes (15 V, 30 min) using a semi-dry transfer (Bio-Rad, Mississauga, ON). Membranes were then blocked (1 hour, RT) with PBS containing 5% milk powder, then incubated with anti-COX-1 (1:2 500), anti-COX-2 (1:2 000), anti-CDC47 (1:16 000), anti-cleaved caspase-3 (1:1 500) or anti-GAPDH (1:50 000) antibody (overnight, 4°C), and subsequently with Horse radish peroxidase (HRP)-conjugated anti-rabbit secondary antibody (1:3 000; RT, 45 min) for the anti-cleaved caspase-3 antibody or with HRP-conjugated anti-Mouse secondary antibody (1:3 000, RT, 45 min) for the anti-COX-1, anti-COX-2 and anti-CDC47 and HRP-conjugated anti-Mouse secondary antibody (1:60 000, RT, 45 min) for anti-GAPDH antibody. Peroxidase activity was visualized with the WestSuper Femto (Pierce), according to the manufacturer's instructions.

### Hoechst staining

Following treatment, both floating and attached cells were resuspended in PBS containing Hoechst 33258 for 24 hours and stored at 4°C. Hoechst nuclear staining was viewed and photographed using a Olympus BX60 fluorescence microscope and a Coolsnap-Pro CF digital Camera (Carsen Group, ON). Cells with typical apoptotic nuclear morphology (nuclear shrinkage, condensation) were identified and counted, using randomly selected fields on numbered photographic slides, of which the "counter" was not aware of the treatment, so as to avoid experimental bias. A minimum of 200 cells per treatment group were counted in each experiment and results are presented as a percentage of apoptotic cells.

### Quantitative real-time RT-PCR analysis

In order to measure the abundance of COX-1, COX-2, EP1, EP2, EP3 and EP4 mRNA, primers were chosen as described below and tested with different primer concentrations and different cycles to avoid mRNA amplification near plateau and saturation. Total RNA (0.2 μg/μl) was used for preparation of first strand cDNA by reverse transcriptase (RT). The RNA samples were incubated (65°C, 10 min) with 2 μl oligo dT (deoxythymidine) primers in a final volume of 10 μl. Samples were then incubated (37°C, 60 min) in 20 μl of a reaction buffer (1X) containing dithiothreitol (DTT; 100 mM), deoxynucleotide triphosphates (dNTPs; 5 mM) and Muloney murine leukemia virus reverse transcriptase (MMLV-RT; 10 U). After cDNA synthesis, the reaction volumes were brought up to 60 μl with autoclaved water. A negative control was also included, using the same reaction mixture but without RNA to ensure absence of any contaminating genomic DNA in the RNA template.

Human COX-1 mRNA was amplified using sense primer 5'-GCAACTGCTTCTTCCCTTTG-3' and antisense primer 5'GGAGTTTGTCAATGCCACCT-3' and human COX-2 mRNA, was amplified using 5'-TGCTTGTCTGGACAACTGC-3' (sense) and 5'-TGAGCATCTACGGTTTGCTG-3' (antisense). For human EP1 mRNA, primers sequences were 5'-TTGTCGGTATCATGGTGGTG-3' (sens) and 5'-ATGTACACCCAAGGGTCCAG-3' (anti-sens), human EP2 mRNA, primers sequences were 5'-TGCTTCTCATGGTCTCGGTG-3' (sens) and 3'-GTGAAAGGCAAGGAGCAGAC-3' (anti-sens), human EP3 mRNA, primers sequences were 5'CAACCTTTTCTTCGCCTCTG-3' (sens) and 5'-TTTCTGCTTCTCCGTGTGTG-3' (anti-sens) and human EP4 mRNA, primers sequences were 5'-GACCTGTTGGGCACTTTGTT-3' (sens) and 5'-TGGACGCATAGACTGCAAAG-3' (anti-sens). Each reaction mixture (final volume, 20 μl) contained RT template or negative control (2 μl), primers (10 μM) and Quantitect SYBRGreen Master Mix (10 μl). Each PCR reaction was inserted in a LightCycler capillary. The quantitative PCR cycling conditions (55 cycles) chosen were (1) 15s at 94°C; (2) 20s at 65°C (COX-1), 63°C (COX-2), 59°C (EP1), 60°C(EP2), 60°C (EP3) and 60°C (EP4); and (3) 11 sec (COX-1), 14 sec (COX-2), 10 sec (EP1), 12 sec (EP2), 15 sec (EP3) and 13 sec (EP4) at 72°C. A melting curve was generated for each reaction and the conditions were (1) 95°C, (2) 30 sec at the annealing temperature, and (3) temperature up to 95°C (0.2°C/sec). Finally, the cDNA concentration of each reaction was determined quantitatively using a standard curve. GAPDH was used as the control reaction.

### PGE2 enzyme immunoassay

The procedure for PGE_2 _EIA kit (Cayman) described by the manufacturer was followed. Briefly, a 50-μl aliquot from culture medium obtained during experimentation was used for PGE_2 _determination in a 96-well plate coated with goat anti-rabbit secondary antibody. A volume of 50 μl of PGE_2 _tracer and 50 μl of the PGE_2 _antibody were added to each sample and the plates were incubated overnight at 4°C. Wells were washed with 10 mM phosphate buffer (pH 7.4) containing Tween 20 (0,05%) at pH 7.4 ; 200 μl of Ellman's reagent (69 mM acetylthiocholine and 54 mM 5,5'-dithio-bis [2-nitrobenzoic acid] in 10 mM phosphate buffer, pH 7.4) was added to each well, and samples were incubated in the dark at room temperature. This allows the bound enzyme tracer to react with Ellman's reagent to yield a yellow solution that can be measured photometrically with a microplate reader at 410 nm. A standard curve was developed simultaneously with standards ranging from 50 to 1000 pg/ml PGE_2_. The presence of PGE_2 _was undetectable in the culture media in the absence of cells.

### Statistical analysis

All experiments were repeated at least six times. Data were subjected to one-way ANOVA or student t test (PRISM software version 4.0; GraphPad, San Diego, CA). Differences between experimental groups were determined by the Tukey's test.

## Authors' contributions

ES drafted the paper and performed experiments. CVT wrote the final version of the manuscript and participated in its design. KL, SP and PL performed some of the experiments. EA conceived the study, participated in its design and coordination, and corrected the final version of the manuscript. All authors read and approved the final manuscript.
